# *Streptococcus intermedius*: an underestimated pathogen in brain infection?

**DOI:** 10.1080/17460913.2024.2423524

**Published:** 2024-11-18

**Authors:** Eliza Gil, James Hatcher, Sophia de Saram, Rebecca L Guy, Theresa Lamagni, Jeremy S Brown

**Affiliations:** a UCL Respiratory, Division of Medicine, University College London, London, WC1E 6JF, UK; b Clinical Research Department, London School of Hygiene & Tropical Medicine, London, WC1E 7HT, UK; c Division of Infection, University College London Hospital, London, NW1 2BU, UK; d Department of Microbiology, Virology & Infection Control, Great Ormond Street Hospital for Children, NHS Foundation Trust, London, WC1N 1EH, UK; e Department of Infection, Immunity & Inflammation, UCL Great Ormond Street Institute of Child Health, London, WC1N 1EH, UK; f Healthcare-Associated Infection & Antimicrobial Resistance Division, UK Health Security Agency, London, NW9 5EQ, United Kingdom

**Keywords:** brain abscess, extradural empyema, streptococcal infections, *Streptococcus anginosus* group, *Streptococcus intermedius*, *Streptococcus milleri* group, subdural empyema, *Viridans streptococci*

## Abstract

*Streptococcus intermedius* is an oral commensal organism belonging to the *Streptococcus anginosus* group (SAG). *S. intermedius* causes periodontitis as well as invasive, pyogenic infection of the central nervous system, pleural space or liver. Compared with other SAG organisms, *S. intermedius* has a higher mortality as well as a predilection for intracranial infection, suggesting it is likely to possess virulence factors that mediate specific interactions with the host resulting in bacteria reaching the brain. The mechanisms involved are not well described. Intracranial suppuration (ICS) due to *S. intermedius* infection can manifest as an abscess within the brain parenchyma, or a collection of pus (empyema) in the sub- or extra-dural space. These infections necessitate neurosurgery and prolonged antibiotic treatment and are associated with a considerable burden of morbidity and mortality. The incidence of ICS is increasing in several settings, with SAG species accounting for an increasing proportion of cases. There is a paucity of published literature regarding *S. intermedius* pathogenesis as well as few published genomes, hampering molecular epidemiological research. This perspective evaluates what is known about the clinical features and pathogenesis of ICS due to *S. intermedius* and explores hypothetical explanations why the incidence of these infections may be increasing.

## Plain Language Summary

*Streptococcus intermedius* is a type of bacteria that normally lives in the mouth. Most of the time it does not cause disease but it can cause mild infections, for example of the gums. Sometimes, it can cause infections deeper in the body, for example the brain, lungs or liver. Brain infection with *Streptococcus intermedius* results in the build-up of pus in the brain or between the skull and the brain. People with these infections need both brain surgery and long courses of antibiotics. Many are left with lasting damage resulting in disability and a significant proportion die from their infection. Several studies have suggested that these infections are becoming more common and that *Streptococcus intermedius* is causing an increasing number of brain infections. Compared with other bacteria that cause these sorts of infections, not much is known about *Streptococcus intermedius* – we do not have many genome sequences or many scientific studies exploring how it causes disease. This piece of work describes what is known about brain infection with Streptococcus intermedius and proposes possible explanations for why these infections may be becoming more common.

## Abstract (plain language summary)

*Streptococcus intermedius*, part of the *Streptococcus anginosus* (formerly milleri) group (SAG), is commensal oral flora but is also able to cause invasive suppurative infection.

*S. intermedius* has a particular predilection for infection of the brain and is associated with a higher rate of abscess formation, higher mortality rate and longer length of hospital stay than other SAG organisms.

The incidence of ICS has been reported to be increasing in several settings.

Streptococci are the dominant cause, other than in post-surgical or traumatic infections, with *S. intermedius* the commonest causative species.

ICS due to SAG organisms is associates with needing a greater number of surgical procedures, increased need for craniotomy/craniectomy compared with other pathogens and longer duration of intravenous antibiotic treatment.

ICS due to *S. intermedius* appears to be increasing in incidence, as are other invasive infections due to SAG organisms. Possible explanations include:
  ○ Increased ability to identify and speciate *S. intermedius*
  ○ Circulation of more virulent and invasive strains of *S. intermedius* h
  ○ Increased rates of *S. intermedius* carriage
  ○ Increased rates, duration or severity of source infections
  ○ Demographic factors, such as aging population and increasing rates of predisposing conditions
  ○ The COVID-19 pandemic, perhaps through altered respiratory viral ecology

There is an apparent in increase in ICS, driven largely by an increase in invasive infection with *S. intermedius*.

The factors underpinning this observed increase are unclear and our ability to appraise them is hampered by our limited understanding of *S. intermedius* as a pathogen.

There is huge scope for future work in this area which is or pressing public health importance.

Key areas for future work include describing the epidemiology of these infections, characterizing the distribution and dynamics of carriage, developing our understanding of the genome and population structure of *S. intermedius* as well as characterizing the organism's virulence factors.

It is time to reappraise the role of this historically undervalued pathogen.

## 1. Introduction

*Streptococcus intermedius* is a microaerophilic Streptococcus and member of the *Streptococcus anginosus* group (SAG), formerly the *Streptococcus milleri* group [1–3]. The SAG is part of what was previously referred to as the viridans group of streptococci (VGS), which also includes the *Streptococcus mutans* group, *Streptococcus salivarius* group, *Streptococcus bovis* group, *Streptococcus mitis* group and the *Streptococcus sanguinis* group [4]. In more recent phylogenetic analyses, SAG organisms have been found to form part of a subclade within the Mitis clade, along with the Pneumoniae, Parasanguinis and Gordonii subclades [5]. In addition to *S. intermedius*, the SAG also includes the species *Streptococcus anginosus* and *Streptococcus constellatus*, with three subspecies of *S. constellatus*: *constellatus*, *pharynges* and *viborgensis*, and two subspecies of *S. anginosus*: *anginosus* & *whileyi* [2,6]. Within the group, *S. intermedius* is more genetically distant relative to the other two species [2,7].

SAG organisms are commensal flora found in the oral cavity, gastrointestinal tract and genitourinary system [8]. All are associated with a range of suppurative infections ranging from superficial infections (particularly periodontal disease, other oropharyngeal infections and rhinosinusitis) to invasive infection resulting in the formation of deep-seated abscesses, often in the brain, lung or liver [8]. SAG organisms cause a notably increased burden of invasive infection in men compared with women [9,10]; the reasons for this remain unclear. Patients partially immunocompromised due to age, diabetes, cirrhosis or malignancy have been identified as at particular risk of invasive infection with SAG organisms [9,11].

Invasive infections with the three organisms show distinct anatomical distributions [8]. *S. intermedius* has a marked predilection for infections of the central nervous system (CNS), head and neck compared with the other SAG organisms. It also causes a considerable burden of infections of the lung and pleural space, skin and soft tissue, as well as the hepatobiliary and gastrointestinal systems [8,9,12–14]. *S. intermedius* appears to be markedly more invasive and more pyogenic than the other members of the SAG, with the highest rate of abscess formation [15]. Conversely, *S. intermedius* is a less common cause of bacteraemia than *S. anginosus* or *S. constellatus* [16], and in experimental models it appears less able to cause infective endocarditis [17]. Significantly, infection with *S. intermedius* is associated with a higher mortality rate and longer length of stay than infection with either *S. anginosus* or *S. constellatus* [1], suggesting it may cause particularly virulent infections.

## 2. Intracranial suppuration

**Figure 1.**
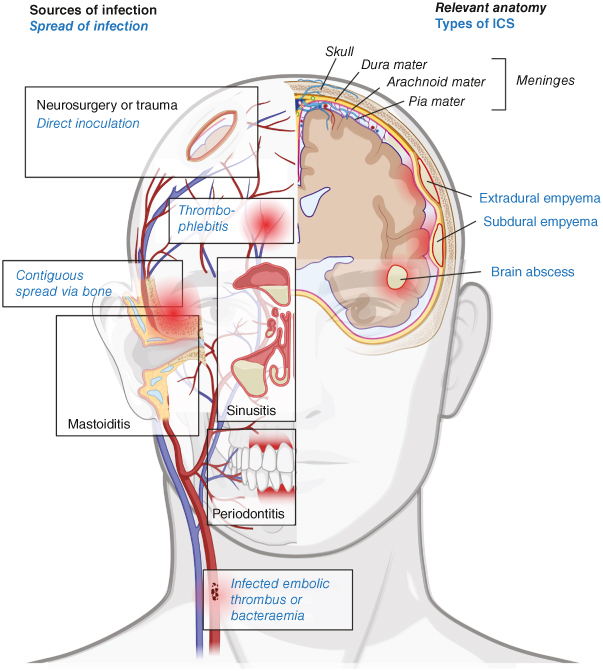
Summary figure of the sources and mechanisms of spread of intracranial suppuration, its types and relevant anatomy, created in BioRender.

ICS occurs in the form of brain abscess or intracranial empyema. A brain abscess is a focal, contained collection of pus within the brain parenchyma [18]. Intracranial empyema is a contained collection of pus occurring either between the skull and the outermost layer of the dura (the dura mater) termed an extradural empyema (or alternatively a cranial extradural abscess), or between the dura mater and the middle layer of the meninges (the arachnoid mater) termed a subdural empyema (Figure 1) [19–21]. Treatment includes initial empiric broad spectrum antibiotics as well as neurosurgical intervention to either aspirate the collection, or to excise parenchymal abscesses with a mature capsule followed by prolonged antibiotic treatment targeting the causative pathogen once isolated [18,22].

The mortality rate of both parenchymal brain abscess and intracranial empyema is typically estimated at between 10 and 20% in adults [23–28], considerably lower than the 50% described historically, which reached 100% in the case of multiple or occipital abscesses [27,29]. The reduction in mortality is likely attributable to improvements in both diagnostic and surgical approaches [30], with the mortality rate reported to still be as high as 45% in patients managed medically [31]. The mortality rate for parenchymal brain abscess is reported to be lower in children, typically reported at between 3 and 5% [32–34].

There are few studies detailing long term functional or neuropsychiatric outcome in parenchymal brain abscess. In most studies addressing this issue, around 30% appear to be left with neurological sequalae [22,24,35]. Notably, rupture of the abscess into the cerebral ventricles is associated with poor outcomes [30]. In subdural empyema, morbidity rates have been reported at 20–55%, with worse prognosis for posterior fossa empyema [20,26,36]. Extradural empyema is less common than subdural empyema but is associated with a lower mortality rate and better prognosis for complete neurological recovery [20].

### 2.1. Epidemiology of ICS

The incidence of brain abscess in recent national-level studies has ranged from 0.76/100,000 in Denmark [23], 1.28/100,000 in England [25] to 1.88/100,000 in Taiwan [37]. In studies examining the incidence over time, the incidence rate of brain abscesses appears to be increasing: in Denmark from 0.6 per 100,000 in 1982-1988 to 0.9 per 100,000 in 2010-2016 [23], while in England the incidence rate has increased from 1.24/100,000 in 1999 to 2.86/100,000 in 2019 [25]. In the US, a marked increase in the incidence of paediatric ICS was observed between 2021 and 2023. There was a baseline median of 34 cases monthly at reporting hospitals between 2016-2019. The monthly case burden was consistently suppressed to below this level during the COVID-19 pandemic. From August 2021 until March 2023 the monthly case burden rose markedly, consistently above the prior median, with a large maximal peak of 102 in December 2022 [38]. This observation has been replicated in smaller scale studies in other settings [39]. The increase in incidence of paediatric ICS has been variously attributed to the effects of the COVID pandemic on viral ecology [40]; however, some data indicates this increase predates the emergence of COVID-19 [25,41]. These data are summarised in Table 1.

**Table 1.** Summary of the epidemiological evidence for the increasing incidence of intracranial suppuration and *Streptococcus anginosus* group infection.

Country (region if applicable)	Included	Quantification	Time period	Ref.
Denmark	All brain abscesses	0.6 cases/100,000 population	1982–1988	[23]
		0.9 cases/100,000 population	2010–2016	
England	All brain abscesses	1.24 cases/100,000 population	1999	[25]
		2.86 cases/100,000 population	2019	
USA	Paediatric ICS	Median 34; IQR: 29.75–42.00, maximum 61, hospitalizations per month at reporting hospitals	2016–2019	[38]
		>40 (no descriptive statistics supplied), maximum 120, hospitalizations per month at reporting hospitals	Sept 2021 – Mar 2023	
England (Liverpool)	Paediatric intracranial empyema	0.3% neurosurgical admissions	Jan 2016 – Dec 2019	[39]
		1.2% neurosurgical admissions	Jan 2020 – Dec 2021	
USA (Texas)	Pediatric brain abscesses associated with sinus or ear infection	All organisms: 2.35 cases/10,000 admissions SAG organisms: 0.94 cases/10,000 admissions	2011	[41]
		All organisms: 7.52 cases/10,000 admissions SAG organisms: 4.4 cases/10,000 admissions	2022	
USA (Texas)	Paediatric SAG infections causing hospital admission	1.9 cases/10,000 hospitalizations	2011	[42]
		16.3 cases/10,000 hospitalizations	2018	

Cases of both brain abscess and intracranial empyema are unevenly distributed across population age groups, occurring most commonly in infants and older adults, in whom the risk increases with increasing age [23,25]. The incidence of both brain abscess and intracranial empyema in England also show a peak in late childhood/adolescence [25]. There is a strong male preponderance for both brain abscess and intracranial empyema at all ages [23–26,37,43–46], with a male to female ratio calculated by meta-analysis at 2.4:1 and as high as 4:1 in some cohort studies [24,46]. The risk of brain abscess also appears to be increased in immunocompromised patients [23].

### 2.2. Pathogenesis of ICS & SAG interactions with the CNS

Three principle mechanisms are implicated in the development of ICS [24], summarized in Figure 1:

- Local spread of infection from elsewhere in the head and neck, typically the nasal sinuses, middle ear or oropharynx, into the CNS.
- Hematogenous spread from distant sites of infection. This can occur in the context of infective endocarditis, but also distant focal infection.
- Secondary to neurosurgery or head injury.

However, many brain abscesses are cryptogenic without evidence of a source. For local sources of infection, infection may spread via two mechanisms (Figure 1):

- Contiguous spread. This involves direct extension of extracranial infection into the intracranial space, typically via local infection spreading to the bone of the skull, causing osteomyelitis, enabling spread to extradural space below. Subsequent penetration of the dura by bacteria can lead to subdural infection, where it can spread due to the lack of anatomical barriers in this space [47].
- Spreading thrombophlebitis, via the facial venous system. The valveless emissary and diploic veins, which connect the extracranial venous system with the intracranial venous sinuses, potentially allow infection to track from extracranial structures into the brain [48]. In addition, the facial and ophthalmic veins, which drain the midface, are in direct communication with the cavernous sinus and, although they have valves, they also provide a direct anatomical connection between extracranial and intracranial vascular structures that could facilitate the spread of pathogens into the CNS [49].

Overall, direct spread of infection, including post-surgical infection, is thought to account for about half and hematogenous dissemination approximately a third of cases, with the remaining cases having no evident source infection [24]. Infection of the sinus or ear are particularly significant sources for subdural empyema [26,27,50–52]. Odontogenic infection, such as decaying teeth or periodontitis, can act as a source infection for parenchymal brain abscess [24,53] and oral injury has also been reported as a source of brain abscess even in the absence of infective endocarditis [54–56]. Conversely, population level data has not demonstrated routine dental visits or dental surgery to be a risk factor for brain abscess [57]. A small proportion of brain abscess and intracranial empyema cases occur secondary to bacterial meningitis, particularly in infants [32,34,58].

The mechanism by which infection at distance sites reach the brain in the absence of a central endovascular source is not well characterized. It is hypothesized that bacteremia can result in bacteria crossing the blood–brain (BBB) or blood–CSF barrier (BCSFB) [59]. In addition, the glymphatic system, which is important for the pathogenesis of meningitis [60,61], is another possible portal for the interaction of pathogens with the CNS via the perivascular spaces. The risk of hematogenous seeding of brain abscesses is increased in the context of structural cardiac or vascular defects causing right-to-left shunts [33,62–66] which allow bacteria in the venous circulation to bypass the pulmonary microvasculature and enter the arterial circulation to facilitate their circulation to the brain. Comparison of the genome of a single isolate of *S. intermedius* with that of *Streptococcus pneumoniae* demonstrated the presence of a complete *cps* locus, which in *S. pneumoniae* encodes the expression of the polysaccharide capsule, a key virulence factor in allowing the survival of the pathogen in the circulation [67].

Crossing the BBB or BSCFB requires either breakdown in the structural integrity of the barrier, which can occur in response to inflammation, or active bacterial translocation across the barrier, which typically relies on interaction of bacteria with specific endothelial receptors [59]. Oral streptococci express antigen I/II that is involved in adhesion to host cells [68], and *S. intermedius* express a putative cell wall AgI/II adhesin called Pas [69]. Pas has sequence homology to BspC in Group B *Streptococcus* (GBS) [69] which is known to interact with host vimentin to promote adherence to brain endothelium during meningitis, as well as inducing neutrophil chemokine expression [70]. Pas has been shown to bind to various host proteins including lung alveolar glycoprotein 340, the extracellular matrix constituents fibrinogen & fibronectin and to platelets [69,71–73]. Hence, Pas is a candidate adhesin required by *S. intermedius* invasion of the CNS; however, there are no data confirming this role as yet. In addition to Pas, *S. intermedius* expresses a fibronectin binding protein, FbpI, which is closely related to a similar molecule in *S. anginosus*, and is involved in adherence to host cells [74]; again, there are no data on whether FbpI is required for adhesion or translocation across brain endothelium. A genomic study comparing *S. intermedius* to other streptococci, which included only 16 *S. intermedius* genomes, also identified expression of known streptococcal adhesins psaA, eno and pulA [75].

### 2.3. Microbiology of ICS

In the majority of cases of ICS at least one bacterial organism is isolated [43,62,76–78]. Streptococci dominate in both parenchymal brain abscesses and intracranial empyema, and are overall the commonest causative pathogen identified by conventional culture techniques [24,26,33,39,43,62,76,77,79,80]. Staphylococci and other skin flora dominate in abscesses and empyema resulting from trauma or neurosurgery [43,81], although studies have demonstrated that many post neurosurgical infections are caused by Gram negative organisms [82]. Interestingly in several older studies, Staphylococci predominates among the Gram positive cocci causing brain abscess of any aetiology, rather than streptococci as commonly reported in more recent studies, despite similar, or lower, rates of abscess secondary to trauma or neurosurgery [29,83].

#### 2.3.1. Polymicrobial infection

Traditional culture techniques have most likely underestimated the proportion of ICS that is polymicrobial, with microscopy of Gram-stained aspirated pus from brain abscesses identifying the presence of diverse bacterial morphology more frequently than culture [43,77]. Metagenomic approaches are addressing this diagnostic challenge and have demonstrated that brain abscesses are often polymicrobial in nature: the proportion that are found to be polymicrobial using these techniques has been reported in several studies at 50–60% [81,84–86]. This is higher in abscesses with a sinus or dental source, where polymicrobial abscesses have been reported to account for between 62.5% (10/16) [85] and 77.8% (7/9) of cases [87]. 16S rRNA sequencing of pus from brain abscesses has demonstrated the significance of the *Streptococcus anginosus* group in this context, alongside other oral obligate or facultative anaerobes: *Fusobacterium spp.*, *Aggregatibacter spp.*, the Parvimonas micra group and *Prevotella spp.* in polymicrobial brain abscesses [81,84–86,88]. In one study, *S. intermedius*, *Aggregatibacter aphrophilus* and *F. nucleatum*, or combinations of them were found in all spontaneous polymicrobial abscesses, with *S. intermedius* identified in 24 out of 42 cases [85].

The presence of anaerobic and facultative Gram-positive cocci, including *S. intermedius*, appears to enhance the growth of many other bacteria [89]. In animal infection models the virulence of anaerobes is increased in the presence of anaerobic streptococci, including *S. intermedius* [90–92]. This positive interaction appears to be bi-directional: in a murine model of orofacial infection, even the heat-inactivated culture filtrate of *Fusobacterium nucleatum* was observed to enhance the virulence of *S. constellatus* [93], although it remains unclear what heat-stable secreted factor mediates this effect. These interactions may drive pathogenesis of polymicrobial ICS, with *S. intermedius* and *F. nucleatum* identified as key initial pathogens for the development of pleural infections by other oral organisms [94,95].

### 2.4. Comparison of clinical features of ICS caused by SAG organisms compared with other bacterial pathogens

Although neither the pathophysiology nor the outcomes of SAG ICS versus non-SAG ICS are well characterized, some data do indicate their maybe significant differences. A cohort study comparing intracranial infection with SAG (88% *S. intermedius*) to non-SAG organisms showed the former were associated with more surgical procedures, typically repeated drainage of collections and an increased need for craniotomy or craniectomy [41]. In another study, rhinosinusitis caused by SAG organisms in children was associated with more intracranial complications and permanent neurologic deficits than other pathogens, and was more likely to require neurosurgical intervention and a longer duration of intravenous antibiotics [96–98]. Interestingly children with acute complicated sinusitis (sinusitis associated with severe infective and complications including ICS) were older than those with another, or no, organism identified, with a median age of 12 compared with 7 years in the non-SAG group [98]. This is particularly noteworthy in the context of the recent peak in ICS incidence among older children/young adolescents in England [25]. Interestingly *S. intermedius* CNS infections are rarely associated with meningitis, with meningitis documented in only 3 of 86 patients with *S. intermedius* CNS infection reported in case reports or case series [99–101].

## 3. *S. intermedius*: an emerging CNS pathogen or a CNS pathogen of emerging importance?

Only a small number of studies have specifically examined the incidence rate of ICS, or invasive infection more broadly, caused by *S. intermedius* or SAG organisms over time, and almost all of these studies are limited to local cohorts rather than national data. A study at Texas Children's Hospital suggested the incidence rate of invasive infections caused by SAG organisms markedly increased between 2011 and 2018 from 1.9/10,000 hospitalizations to 16.3/10,000, with a particularly marked increase from 2017 to 2018 (5.9 to 16.3 cases/10,000 hospitalizations) [42]. Even disregarding 2018, the trend of increasing hospital admissions due to SAG infection remained significant [42]. There were increase in all disease categories studied, but the main contributor to the overall increase were ICS, increasing from 0.94 to 8.15/10,000 hospitalizations and mastoiditis, increasing from 0 to 2.39/10,000 hospitalizations [42]. Similar data were obtained at the Children's Hospital Colorado with admissions for acute complicated sinusitis due to SAG organisms rising 15% per year between 2010 and 2016 [98], and in Queensland Australia where there was an 29% annual increase in SAG-associated intra-abdominal infection per 1000 hospitalisations between 2009 and 2019, although this was not seen in other infection types in this study [14].

In the Texas Children's Hospital study, which looked at all invasive infections with SAG organisms, *S. intermedius* was the most commonly isolated SAG pathogen, identified in 80% of cases, followed by *S. constellatus* (12.6%), then *S. anginosus* (7.4%), with a similar breakdown for ICS cases alone [42]. At the Children's Hospital Colorado the proportion of cases of acute complicated sinusitis in admitted children caused by SAG organism rose from 4/22 (18%) in 2010 to 16/27 (59%) in 2016, and SAG organisms were over-represented among children with intracranial complications [98].

The above data suggest that ICS due to *S. intermedius* is increasing in incidence, as are other invasive infections caused by SAG organisms. This could hypothetically be caused by increased rates of identification and/or speciation of the organism (i.e. artefactual), a true increase in virulence of SAG organisms, including increasing rates of antimicrobial resistance (AMR), changes in host factors which can increase the rates of carriage or rates of predisposing infections (possibly including the COVID pandemic), or changes at a population level that might affect rates of predisposition to infection. These possible contributory factors are all explored below.

### 3.1. Under-identified means undervalued?

Demonstrating that a case of ICS is caused by a SAG organism requires microbiological techniques that are able to accurately speciate SAG isolates from other streptococci, especially other member of the VGS. However, SAG organisms are difficult to speciate phenotypically, with overlapping and variable patterns of haemolysis, Lancefield grouping and sugar fermentation [3,102]. Historically, this led to SAG organisms typically being reported only as VGS, or as *S. anginosus* or *S. milleri* group [33,66,76,77,103–105]. Matrix-assisted laser desorption ionization-time of flight mass spectrometry (MALDI-ToF), a more recent approach to microbiological identification, did not originally readily speciate VGS isolates [106–108], with a specific weakness in identifying *S. intermedius* [7,109,110]. Updated databases have considerably improved speciation, although again few SAG organisms were included in the relevant studies [111,112]. 16S PCR is able to speciate the group, resulting in greater ability to specifically identify these pathogens in clinical specimens in centres with access to this technology [113]. The type of sampling performed is also important to the likelihood of isolating SAG organisms, with *S. intermedius* identified much more frequently from intracranial samples compared with sinus samples in children with ICS in the context of complicated rhinosinusitis [114]. When oral streptococci have been speciated accurately, the significant role of *S. intermedius* both in monomicrobial or polymicrobial infection ICS cases has become more apparent [81,88,115,116]. In a recent UK study, SAG accounted for 69% (27/39) of brain abscesses with a confirmed microbiological diagnosis, with *S. intermedius* accounting for 70% (19/27) of these [115]. In a case series of paediatric intracranial empyema, *S. intermedius* was found to be the dominant causative pathogen [39,41], with *Streptococcus pneumoniae* and *Streptococcus pyogenes*, which in other contexts are considered more virulent species, both being identified only infrequently [117,118].

Overall, it is likely that a proportion of the observed increase in SAG ICS cases reflects increased rates of identification. However, MALDI-ToF has been commercially available since the early 1990s and its widespread clinical use in resource-rich settings predates the increase in SAG ICS, suggesting better case ascertainment is unlikely to fully account for the observed increase.

### 3.2. Increasing virulence?

The observed increase in *S. intermedius* ICS could be related to the recent circulation of more invasive strains related to specific virulence factors. Although overall the virulence of SAG organisms is very poorly understood, there are some described virulence factors that conceivably could underpin an increase in virulence for particular SAG strains. These include an array of hydrolytic enzymes [119–125] produced by *S. intermedius* which drive tissue destruction, and the pore-forming cholesterol-dependent cytolysin (CDC) toxin intermedilysin (ILY) which is cytotoxic [126,127]. Both hydrolytic enzymes and ILY could play a role in the establishment of invasive infection and formation of deep-seated abscesses. For example, ILY expression was demonstrated to be 6- to tenfold higher in *S. intermedius* isolates from deep-seated infections, including brain abscesses, compared with carriage, soft tissue abscess, or sinusitis isolates [128]. ILY expression is regulated by two transcriptional repressors, CcpA and LacR [129,130]. LacR mutations resulting in a loss of repression, and therefore higher ILY expression, were identified in *S. intermedius* isolates from invasive infection, supporting a role for increased ILY expression in augmenting the virulence phenotype of *S. intermedius* [130]. However, other SAG organisms do not seem to express ILY or an alternative CDC [128]. As the incidence of invasive infection with all three species appear to be increasing, this makes it less likely that increased ILY toxin expression alone has driven the increase in invasive *S. intermedius* infections. At present, there are no studies exploring the comparative virulence of *S. intermedius* or SAG strains, and the available genome data is too limited to identify whether recent infections are caused by strains from different lineages compared with historical infections. Future work is needed to explore whether recent *S. intermedius* strains causing ICS belong to specific subclades associated with phenotypes that are potentially pathogenic. This will also require a better understanding of the repertoire of virulence determinants for ICS caused by *S. intermedius* and SAG organisms.

### 3.3. Increasing carriage?

An increase in the prevalence of carriage of *S. intermedius* could play a role in the increase in invasive infections with this organism. Widespread use of pneumococcal conjugate vaccine (PCV) is associated with alterations in the nasopharyngeal microbiome, including a reduced presence of typical commensal species [131], increased diversity [132,133] and increased abundance of non-pneumococcal streptococci [133]. These effects could potentially affect the prevalence of carriage of SAG organisms, but many of the studies exploring the effect of PCV on the nasal microbiome were unable to speciate streptococci and so did not examine SAG carriage. Introduction of PCV has altered the repertoire of pathogens causing acute otitis media and bacterial rhinosinusitis, suggesting that it is plausible that a similar effect has increased the incidence of *S. intermedius* infections [134–136]. However, the rate, temporal dynamics and demographics of oro- or naso-pharyngeal carriage with *S. intermedius* is extremely poorly characterized and there is currently no evidence to support whether changes in colonisation rates (potentially related to introduction of PCV) underpins the increase in ICS caused by this organism. Future work is needed to characterize the rates and temporal dynamics of *S. intermedius* carriage across populations, as well as exploring whether this has been affected by the widespread use of PCV.

### 3.4. Increasing rates, duration or severity of source infections?

As already described above, most cases of ICS are the result of local spread from an extracranial infection, typically of the oropharynx, nasal sinuses or middle ear/mastoid. Increases in incidence, duration or severity of these source infections could hypothetically be underlying observed increases in downstream CNS infections. For example, worsening dental health, accompanied by an increase in periodontal infections, could drive a corresponding increase in the incidence of ICS caused by *S. intermedius* [137]. However, the incidence of ICS infections caused by *S. intermedius* or other SAG organisms have increased up to fourfold between 2011 and 2022 [41], and it seems implausible that this reflects a similar increase in the rates of common predisposing milder infections of the sinuses, ears, or oral cavity. Furthermore, in a recent UK study *S. intermedius* and other SAG organisms were particularly dominant causes of brain abscesses for which no primary source of extra-cranial infection were identified (74.2% of cases, compared with 33.3% of cases with an extracranial infective source) [138]. This suggests that even if increasing rates, severity or duration of potential source infections were contributing to the increasing incidence of *S. intermedius* ICS this is unlikely to be the whole picture.

Another factor that could contribute to the increase in *S. intermedius* ICS is reduced use of antibiotics for upper respiratory tract infections as part of antibiotic stewardship initiatives. There is no evidence to firmly support or refute this hypothesis. An additional important factor that could be playing a role in an increase in *S. intermedius* ICS is increased AMR, which might impact the efficacy of treatment for source infections. SAG organism can acquire beta lactam resistance through penicillin-binding mutations, including to third-generation cephalosporins [139], which would be of considerable significance in the context of ICS. Studies which have looked at antibiotic resistance in SAG have so far found no evidence for emerging resistance to beta-lactam antibiotics with only low rates of resistance to other classes of antibiotics [140–142]. Many of these studies included non-invasive, oral isolates and may not be representative of the organisms causing ICS; interestingly a recent study of *S. intermedius* invasive infection in children demonstrated high rates of resistance to macrolides (73.3%) and clindamycin (76.7%); all isolates were sensitive to third generation cephalosporins but 7.4% were penicillin resistant [12].

Future work to explore the rates of secondary complication of upper respiratory tract infections is needed but is challenging as these infections are exceedingly common, often do not lead to contact with healthcare services, and secondary complications such as ICS are exceedingly rare.

### 3.5. Other demographic factors

Neurosurgery is a significant predisposing factor for the acquisition of ICS, but is much less important for the pathogenesis of ICS caused by SAG organisms, with only a single case report of *S. intermedius* ICS caused by neurosurgery [143]. Therefore, increasing rates of neurosurgery can be discounted as a causative or contributory factor to the increasing incidence of *S. intermedius* ICS. Other host factors that predispose to both invasive infection with SAG organisms and ICS more broadly include age, diabetes, cirrhosis of the liver, immunosuppression and malignancy [9,11,23–25]. The aging population makes these comorbidities commoner and so potentially contributing to the increased incidence of *S. intermedius* ICS. However, the increase in *S. intermedius* ICS in England appears to be occurring across all ages rather than concentrated in older adults alone, with a marked spike in late childhood/early adolescent that is unlikely to be attributable to increases in age-related comorbidities. Future work is needed to better characterize the disease burden of ICS as a whole, as well as that specifically due to *S. intermedius* and the other SAG organisms, across the population, characterising the contribution of predisposing conditions.

### 3.6. A role for COVID-19?

In the US, a marked increase in the incidence of paediatric ICS was observed between 2021 and 2023 [38], with a specific increase in the burden of SAG ICS since the COVID-19 pandemic [144]. This observation suggests there may be a relationship to the COVID-19 pandemic. The introduction then lifting of non-pharmaceutical interventions to limit the spread of COVID-19 is known to have had a profound effect on upper respiratory tract infections. For example, relaxation of non-pharmaceutical interventions was associated with an increased incidence of mastoiditis [145], which could have led to an increase in downstream intracranial infections. Furthermore, there were profound alterations in the epidemiology of respiratory viruses [146] which are known to predispose to subsequent secondary bacterial infection [147], possibly including ICS by *S. intermedius* or SAG organisms. However, while the COVID-19 pandemic might have accelerated or exposed the trend of increasing ICS, the data indicates the incidence of these infections was increasing for at least a decade [25,41] and therefore well before the 2020 COVID-19 pandemic.

## 4. Conclusion

The incidence of *S. intermedius* ICS appears to be increasing in diverse settings, both geographically and across age groups. These infections are associated with a significant burden of mortality and morbidity, with *S. intermedius* ICS associated with the need for multiple neurosurgical procedures and prolonged intravenous antibiotics. There is also evidence that other invasive infections caused by SAG organisms are becoming more common. The factors underpinning these observations are unclear. Hypothetical contributory factors could include better case ascertainment through increased ability to speciate these organisms, the circulation of more virulent strains, increased rates or carriage or altered microbial ecology in the oropharynx, an increasing rate, duration or severity of source infections, altered population demographics or downstream effects of the COVID-19 pandemic. SAG organisms are extremely poorly characterized compared with other streptococcal pathogens, hampering our ability to unravel the relative contributions made by each of these. There is a pressing need for work to better characterize the burden of these infections and to explore the factors contributing to their increase.

## 5. Future perspective

This article has highlighted an apparent increase in ICS, which appears to be largely due to an increase in invasive infection with *S. intermedius*. However, there are extensive knowledge gaps undermining our ability to characterize the reasons for this increase. The nature and burden of invasive infection with SAG organisms is poorly characterized, including minimal description of the burden of SAG ICS at anything above the local level. While a number of studies have provided some insights into the epidemiology of diseases due to SAG, further explorations are needed to understand the populations at risk and look for evidence of clustering of disease. There is no described phylogenetic population structure for *S. intermedius* and only a limited number of published genome sequences, undermining our ability to conduct molecular epidemiology to link cases or discern whether the increase is caused by a specific strain or clade. Another significant current knowledge gap is the lack of understanding of SAG carriage, including the temporal dynamics and demographics of patients colonised with SAG strains. Without understanding carriage, it is impossible to address whether alterations in the carriage rate, perhaps secondary to the widespread use of PCV, are contributing to an increase in SAG ICS. The virulence factors of *S. intermedius*, and the other SAG organism, are also extremely poorly characterized compared with other streptococcal pathogens. If the increase in cases of SAG ICS is being driven by increased virulence of the organism, our limited understanding of SAG virulence will hamper future exploration of whether changes in circulating clades has resulted in higher exposure to more virulent organisms and therefore increased the incidence of invasive infections such as ICS.

Overall, there is huge scope for future work in this area, much of which is of pressing public health importance. It is key that larger scale epidemiological work is undertaken to characterize the incidence and demographics of ICS due to *S. intermedius*, and whether this has changed over time. The roles of comorbidities and source infections also need to be specifically explored, particularly whether these are contributing to the markedly unequal incidence between males and females. Developing our understanding of the genetic variability and population structure of *S. intermedius* will be imperative to assess whether any specific strains or clades are associated with ICS and therefore of particular public health concern. Carriage of *S. intermedius* needs to be characterized across different populations, including the temporal dynamics of carriage and whether this has changed, or is changing, over time. It would also be of interest to explore the specific impact of PCV on the carriage of *S. intermedius*.

With regards to the pathogen itself, both the genomic and phenotypic determinants of its virulence need to be better characterized. Key questions include what mechanisms *S. intermedius* uses to evade the circulating host immune response, how does it cross the cerebrovascular barrier and the role of secreted factors during pathogenesis, notably intermedilysin toxin and degradative enzymes. As metagenomic technologies and more advanced diagnostic microbiological techniques become increasingly available, it will become easier to characterize polymicrobial infections. This will hopefully facilitate future work to elucidate the interplay of *S. intermedius* with other key oral anaerobes in the establishment of ICS.

The burden of ICS appears to be increasing in diverse populations at disparate geographic locations, with the majority of cases caused by *S. intermedius*. It is time to reappraise the role of this historically undervalued pathogen, along with its fellow SAG members, in pyogenic infection. While ICS remains rare, given the heavy burden of morbidity and mortality there is a pressing need to further our understanding of this condition including the role of *S. intermedius* to underpin the design of potential future surveillance and interventive measures.
